# Universal Triboelectric Nanogenerator Simulation Based on Dynamic Finite Element Method Model

**DOI:** 10.3390/s20174838

**Published:** 2020-08-27

**Authors:** Jinkai Chen, Junchao Wang, Weipeng Xuan, Shurong Dong, Jikui Luo

**Affiliations:** 1 Ministry of Education Key Lab of RF Circuits and Systems, College of Electronics & Information, Hangzhou Dianzi University, Hangzhou 310000, China; junchao@hdu.edu.cn (J.W.); xuanweipeng@hdu.edu.cn (W.X.); jackluo@zju.edu.cn (J.L.); 2 Key Lab of Advanced Micro/Nano Electronic Devices & Smart Systems of Zhejiang, College of Information Science & Electronic Engineering, Zhejiang University, Hangzhou 310000, China; dongshurong@zju.edu.cn

**Keywords:** triboelectric nanogenerator, finite element model, dynamic behaviors, electrostatic induction

## Abstract

The lack of a universal simulation method for triboelectric nanogenerator (TENG) makes the device design and optimization difficult before experiment, which protracts the research and development process and hinders the landing of practical TENG applications. The existing electrostatic induction models for TENGs have limitations in simulating TENGs with complex geometries and their dynamic behaviors under practical movements due to the topology change issues. Here, a dynamic finite element method (FEM) model is proposed. The introduction of air buffer layers and the moving mesh method eliminates the topology change issues during practical movement and allows simulation of dynamic and time-varying behaviors of TENGs with complex 2D/3D geometries. Systematic investigations are carried out to optimize the air buffer thickness and mesh densities, and the optimized results show excellent consistency with the experimental data and results based on other existing methods. It also shows that a 3D disk-type rotating TENG can be simulated using the model, clearly demonstrating the capability and superiority of the dynamic FEM model. Moreover, the dynamic FEM model is used to optimize the shape of the tribo-material, which is used as a preliminary example to demonstrate the possibility of designing a TENG-based sensor.

## 1. Introduction

Scavenging environmental mechanical energy using triboelectric nanogenerators as energy sources [1,2,3] or self-powered sensors [4,5,6,7,8,9,10,11] for future maintenance-free applications has attracted much attention since the invention of the triboelectric nanogenerator (TENG) in 2012 [12]. The operating principle of TENGs is the combination effects of contact electrification and electrostatic induction. An in-depth understanding of these processes is essential for the design of high-performance TENGs and will help to promote practical applications of the TENGs.

**Figure 1:**
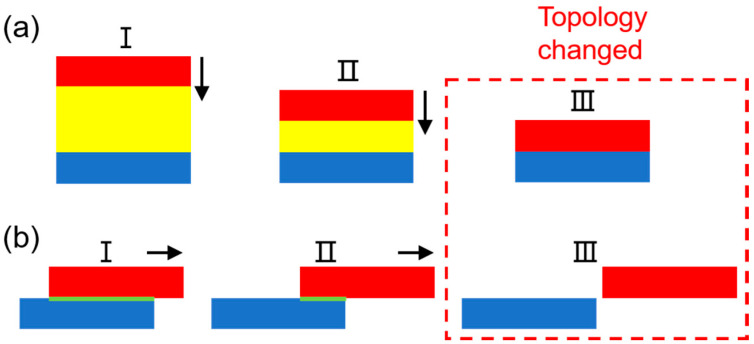
Practical movement of contact and sliding mode TENG makes the FEM geometry have topology change issues. (**a**) The topology of contact mode TENG changes due to the vanish of air layer (yellow) when upper tribo-material (red) moving from state I to III. (**b**) The topology of sliding mode TENG changes due to the vanish of the overlapped boundary (green) when upper tribo-material (red) moving from state I to III.

Various models for TENGs have been proposed and explored to explain the charge transfer process during the contact of two tribo-materials (i.e., the contact electrification) such as electron-cloud model [13,14,15], ion-transfer model [16], material-transfer model [17], etc. No matter which model is applied, a constant surface charge density is always assumed to quantitatively represent the transferred charges remaining on the surfaces of the tribo-materials, even after contact electrification. The surface charge density can be theoretically calculated using first-principle approaches [18,19]. However, from the contact electrification-based simulation, it is impossible to predict the charge transfer in the external circuit. Different from this, the electrostatic induction process reflects the redistribution of induced charges on the back electrodes of TENG, as well as the charge transfer process between the external circuit and back electrodes, rather than triboelectric charge (i.e., surface charge density on the surface of tribo-materials). The charge redistribution is strongly related to material properties, device structure and operational parameters such as movement, contact force of the tribo-materials. As such, analytical [20,21,22] and numerical [23,24,25] methods based on the electrostatic induction process have also been developed and successfully applied for TENGs of different working modes such as the contact mode [20], sliding mode [23], freestanding mode [26], etc., but they are limited to some simple cases. The existing methods have limitations in calculating complex cases, and the problems associated include the following:

(1) Analytic methods can easily obtain accurate dynamic voltage/current outputs (i.e., I(t) or V(t)) for some specific operation mode TENGs with simple geometries, but can hardly deal with the sliding mode TENG, or TENGs with complex 2D or 3D geometries.

(2) Numerical methods can easily simulate contact/sliding mode TENGs with complex 2D/3D geometries, but the achievable results are mostly limited to steady state potential distribution. Although some recently developed numerical methods are capable of simulating the time-varying behavior of TENGs with 3D geometries with limited movement range (i.e., two tribo-materials should not be fully separated from each other) [24,25], they cannot simulate dynamic voltage/current outputs of TENGs under practical movements (i.e., tribo-materials are fully separated) due to the topology-change problems. The topology-change issues in contact and sliding mode TENGs are highlighted in Figure 1. It is clear in Figure 1a that the air layer (yellow) vanishes (i.e., topology changed) when the upper tribo-material (red) finally contacts with the lower tribo-material (blue); for the sliding mode TENG, the overlapped boundary (green) also vanishes when the upper tribo-material (red) moves from state I to III as shown in Figure 1b.

Although the existing models are useful for partially understanding the principle/operation of TENGs, the lack of an adequate approach for simulating the time-varying and dynamic behaviors of TENGs with complex geometries under practical movements prevents in-depth understanding of TENGs in operation and further development of high-performance TENGs or TENG-based sensor devices.

Here, we focus on the research of electrostatic induction for TENGs, and propose a novel universal numerical method which is capable of calculating dynamic behavior of TENGs with complex 2D/3D geometries under practical movements using finite element method (FEM). By introducing a moving mesh method and air buffer layers in TENG simulation, the unavoidable topology-change issues in the existing simulation models while dealing with practical movements can be eliminated. Thus, it is possible to calculate the dynamic behavior of 2D/3D TENG geometries. The universal dynamic FEM numerical model is extremely beneficiary for the structural design of TENGs with practical experimental setup, combined with the ongoing in-depth research of the contact electrification, are expected to promote practical TENG applications.

## 2. General Theory

The key problem for simulating time-varying behaviors of 2D/3D TENGs with practical experimental configuration using the FEM method is to solve the topology-change issues. In this research, we start with the most fundamental working modes (i.e., contact mode and sliding mode) for TENGs, the solution of which can be easily applied to other working modes such as the single electrode mode and freestanding mode. We first introduce the well-known general theory for dynamic TENG simulation based on FEM, then focus on how to solve the topology-change problems.

The finite element method divides the TENG 2D/3D geometries into many basic elements (i.e., the meshing process), and each mesh element follows the same constitutive equations, which is based on Maxwell’s displacement current. The basic simulation flow for time-dependent TENG FEM model is similar to other researches [24,25], thus, we briefly introduce it here. The relationship between displacement field (**D**) and charge density (*ρ*) is described by the Maxwell–Poisson equation,

(1) ∇·D=ρ

It should be noted that equation 1 is applicable to both tribo-material and back electrode; here, *ρ* represents the charge density of free charges on the back electrode as well as the triboelectric charges in the tribo-material. For a displacement field (**D**), it can be related to the electric field (**E**) using the following equation in an isotropic media:

(2) $$D=\varepsilon_{r}\varepsilon_{0}E$$

where *ε_r_* and *ε*_0_ represent permittivity of the dielectric material and vacuum, respectively. In the absence of magnetic field, the electric field (**E**) can be derived as follows:

(3) E=−∇ϕ

where *ϕ* represents the electric potential in the tribo-material. Combining Equations (1)–(3), the relationship between electric potential and charge density can be finally derived,

(4) $$-\varepsilon_{r}\varepsilon_{0}\nabla^{2}\phi =\rho$$

Based on the above electrostatic constitutive equation, the link between electric potential and surface charge density is established. The electric potential can be numerically obtained by combining the above equations with the moving meshes and boundary conditions, which will be detailed in the subsequent sections as the configurations for the contact and sliding modes are quite different.

## 3. Results

### 3.1. Dynamic Behavior of Contact Mode TENGs

**Figure 2:**
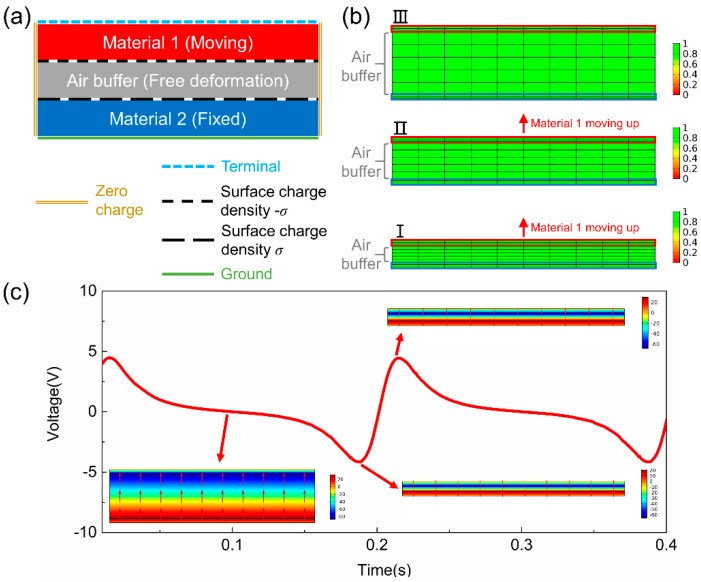
(**a**) Boundary conditions for contact mode TENG. (**b**) Mesh remains best quality when contact mode TENG is moving from state I to III. (**c**) The voltage potential distribution and the dynamic voltage output under a 10-MΩ external load.

The numerical calculation is carried out using COMSOL software. The boundary conditions for the contact mode TENG are shown in Figure 2. The triboelectric surface charge densities on the contacting surfaces of Materials 1 and 2 are *σ* and −*σ*, respectively. The back electrode of Material 2 is set as the ground (i.e., voltage equals to 0), and the back electrode of Material 1 is set to be the terminal for current to flow to the external circuit, which is governed by the Kirchhoff’s law. The output current (*I*) can be mathematically expressed as follows:

(5) $$\int_{s}D\cdot ndS=Q_{free};\;I=\frac{dQ_{free}}{dt}$$

where *S* and **n** represent the back-electrode surface of Material 1 and the normal vector perpendicular to it, respectively. By integrating the normal component of electric displacement field over the back-electrode surface, the free charge *Q_free_* can be derived. Similarly, by differentiating it over time, the output current *I* can be obtained. The rest of the boundaries are set as zero charge. Combining with the electrostatic and electrical relation formulas, both the voltage potential distribution and the dynamic voltage/current outputs can be simulated, and the power output under different external loads can also be derived.

The FEM model divides the TENG 2D/3D geometries into many basic domains, and for calculating the time-varying performance of a contact mode TENG, some of the meshed domains in TENG need be deformed to simulate the cyclic moving process. However, the mesh topology must be kept unchanged, i.e., no mesh elements should be eliminated or newly created, for convergency of the FEM solver. To solve this problem, an air buffer layer is introduced in the model as shown in Figure 2a, and the thickness of which will not shrink to zero for an unchanged topology. The moving mesh method is used for the moving mesh nodal points throughout the region to best approximate the updated geometry boundaries. The boundary conditions for the moving mesh method are also illustrated in Figure 2a. The mesh inside the area of Material 2 is fixed. The mesh in Material 1 is moving as a whole and the relative positions of mesh nodal points in Material 1 remain unchanged. For mesh in the air buffer layer, they will shrink or expand in accordance with the motions to keep the mesh topology unchanged and to simulate the motion process of the contact-separation mode TENG, thus, the model provides a free deformation configuration during simulation. The movement of the mesh nodal points inside the air buffer layer is governed by the following equation over time:

(6) $$\frac{\partial^{2}}{\partial X^{2}}\frac{\partial r}{\partial t}+\frac{\partial^{2}}{\partial Y^{2}}\frac{\partial r}{\partial t}+\frac{\partial^{2}}{\partial Z^{2}}\frac{\partial r}{\partial t}=0$$

where **r** represents absolute coordinates of the mesh nodal points, and *X*, *Y*, *Z* represents the reference coordinates between mesh nodal points inside the air buffer layer.

The moving mesh in the air buffer area at different operation times is illustrated in Figure 2b. The large red and blue rectangles represent Materials 1 and 2, respectively. Evenly mapped meshes are applied to all the areas of TENG. It can be seen that the mesh in the air buffer layer expands vertically when Material 1 moves from state I to III, while the number of mesh nodal points remains unchanged. The color bars on the right side represent the mesh element quality with the scale varying from 0 to 1, with 1 meaning the best mesh quality. Combining the moving mesh results with the electrostatic theory mentioned above, the voltage potential distribution and the dynamic voltage/current output can be calculated simultaneously, which is shown in Figure 2c with a 10-MΩ external load. The color legends represent the electric potential. It can be easily summarized from Figure 2c that the largest electric potential occurs when two tribo-materials separate the most at ~0.1 s, while the voltage output in the external circuit decreases to zero voltage at ~0.1 s. As Material 1 approaches Material 2 at 0.189 s, the electric potential in TENG decreases, but the voltage output reaches the negative maximum. After that, Material 1 contacts Material 2 and starts separation, which leads to a maximum voltage output at 0.215 s. It should be noted that the voltage output needs a relaxation time to reach a steady state as the external load hinders the charge transfer, and a larger external load leads to a longer relaxation time.

**Figure 3:**
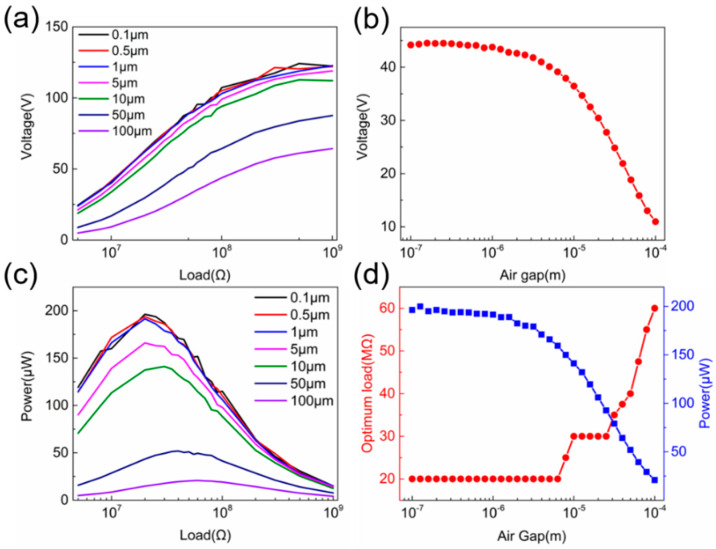
Simulated (**a**) voltage and (**c**) power output when the thickness of air gap increases from 0.1 to 100 μm; (**b**) summarized peak voltage at a 1-MΩ external load with increasing air gap thickness; and (**d**) summarized peak power and correspondence optimum load under increasing air gap.

Optimizations are carried out for the air buffer thickness and mesh density. The simulation parameters used during optimization are listed in the Supplementary Information, Table S1. As the mesh topology must remain unchanged throughout the simulation, the air buffer layer cannot be shrunk to zero, which differs slightly from real experimental configuration; therefore, the starting thickness of the air buffer layer is set to a value close to zero but not zero. Using Table S2 as an example, it is clear that a thicker air buffer layer will lead to a much shorter calculating time, but a worse consistency between experimental and simulated results, and vice versa. Thus, an optimal air buffer thickness should be determined first for our model. The voltage and power outputs with various external loads using different air buffer thicknesses are simulated, with the results shown in Figure 3a,c, respectively. The summarized peak voltage at a 10-MΩ external load is shown in Figure 3b, which remains almost unchanged (<1% discrepancy) when the thickness of the air buffer layer is less than 1 μm (1/100 of tribo-material thickness). If a 10% voltage discrepancy can be tolerated, then the thickness of the air buffer layer of ~5 μm can be used for simulation to save a lot of computing time, which is around 1/20 of the tribo-material thickness. The peak power and corresponding optimum load are shown in Figure 3d, which experiences a similar trend as that of the voltage output. The peak power and optimum load remain almost unchanged for the air buffer layer thickness range from 1 to 6.3 μm. The thickness of the air buffer layer can further be increase to ~3.5 μm to reduce the computation time if a 10% voltage discrepancy can be tolerated.

**Figure 4:**
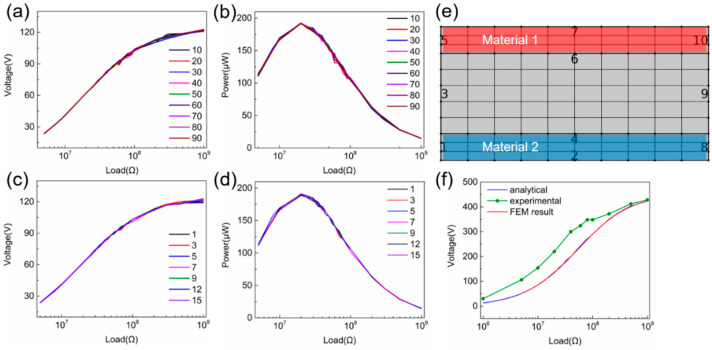
Simulated (**a**) voltage and (**b**) power output with increasing mesh density in horizontal boundaries; simulated (**c**) voltage and (**d**) power output with increasing mesh density in vertical boundaries. (**e**) The mapped mesh of contact mode TENG with vertical (1, 3, 5, 8, 9 and 10) and horizontal boundaries (2, 4, 6, and 7). (**f**) Comparison of experimental data and simulated results based on the dynamic FEM model, analytical model.

In most cases, the mesh density can affect the simulation accuracy significantly, but it is not practical to increase mesh density infinitely due to the limited computational capability. The optimal mesh density is investigated for the contact mode TENG, and the voltage and power outputs under different loads using various mesh densities are shown in Figure 4. As mentioned before in Figure 2b, mapped meshes are applied to all the areas of TENG, which divides the TENG geometry into many rectangular areas, and the mesh density is determined by the vertical and horizontal boundaries as shown in Figure 4e. Figure 4a,b shows that the simulated voltage and power peak outputs remain nearly identical when the mesh nodal points in the horizontal boundaries (2, 4, 6, and 7) increase from 10 to 90, and the same trend occurs when the mesh nodal points in the vertical boundaries (1, 3, 5, 8, 9 and 10) increase as shown in Figure 4c,d. A higher mesh density will only lead to less output curve distortion. This trend can be attributed to the highly symmetric geometry of the contact mode TENG model. Combining with the symmetric mesh, the mesh quality remains at the highest rate of 1 and larger mesh density will not improve the simulation accuracy significantly. Thus, a moderate mesh density should be chosen for less curve distortion. Based on the systematical investigation above, optimal parameters can be obtained for the contact mode TENGs. For the air buffer layer, the optimum thickness is about 1 μm. For the vertical mesh density, the optimum density is about 50 with even distribution. As for the horizontal mesh density, three material areas have different mesh numbers; the optimal mesh densities are about 5, 10, 25 for Material 1, the air buffer, and Material 2, respectively.

To verify the dynamic FEM model for the contact mode TENG, the simulated results based on the dynamic FEM model are compared with the experimental data and analytical simulated results based on our previous work [27], as shown in Figure 4f. The simulation parameters for the dynamic FEM model and analytical model are listed in the Supplementary Information, Table S3. The practical movement was extracted from the dynamic fatigue tester (Popwil Instrument, Hangzhou, China, Model YPS-1) [27], which is listed in the Supplementary Information, Table S4. The experimental voltage output was measured using an oscilloscope (Tektronix, Beaverton, OR, USA, MDO3022) with a 100:1 voltage probe, and one of the electrodes of TENG is grounded due to the voltage probe. The calculated voltage under different loads using the dynamic FEM and analytical model is highly identical, which is reasonable as the basic constitutive relations are exactly the same. Both of them are close to the measured voltage output of the TENG, clearly demonstrating that the dynamic FEM model can be used to calculate the contact mode TENG accurately. The discrepancy between experimental and FEM/analytical results can be attributed to three reasons. Firstly, the simulation parameters extracted from experiments are not perfectly accurate. Secondly, the experimentally measured voltage output curve under a low external load can be very unstable, which leads to much larger discrepancy under a relatively lower eternal load. Lastly, the analytical and FEM results are calculated under the bases that the electric potential has a uniform distribution horizontally. Since the length and width of the tribo-plate are much larger than the maximum displacement in vertical, the TENG is normally treated as a device with two infinite plates with a uniform electric potential between the plates. However, a faint edge effect will still exist in experimental results, which also leads to a discrepancy of the experimental and FEM/analytical results.

### 3.2. Dynamic Behavior of Sliding Mode TENG

Owing to the symmetric geometry of contact mode TENGs, the mesh quality remains very high during movements. However, the case for sliding mode TENGs is quite different due to its asymmetric geometry, thus needs different geometry, meshing method, boundary conditions and optimizations to solve the topology-change issues for accurate simulation. For the existing simulation methods for sliding mode TENGs, the general approach is to assume two materials contact directly with each other and only the non-overlapped areas have surface charge [23,25]; additionally, during simulation, the two tribo-materials must not be totally separated from each other as disappearance of the overlapped areas will result in the topology-change issue as well as the convergence problems. Therefore, a new approach is needed to simulate sliding mode TENGs, particularly the rotating (sliding) mode TENGs, as the moving tribo-material in sliding mode TENGs is periodically totally separated from the fixed one. Similar to what we used in the contact mode case, here we introduce the moving mesh method and air buffer layers for sliding mode TENG simulation.

**Figure 5:**
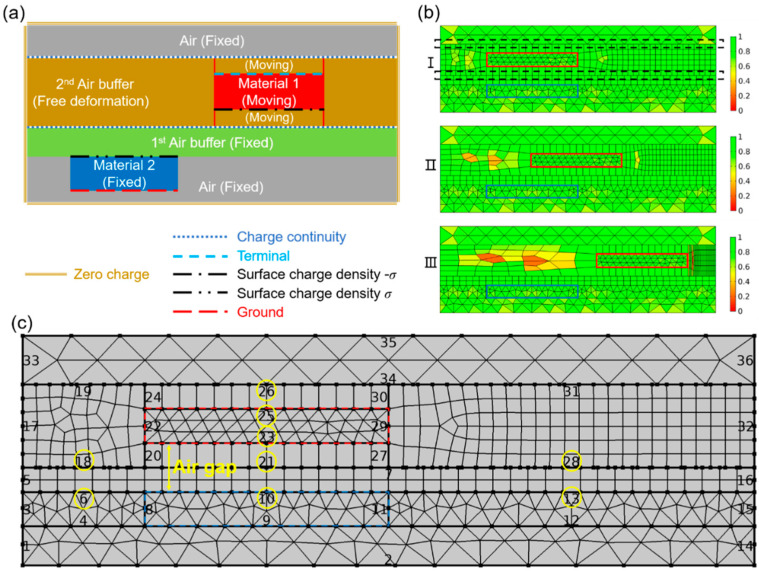
(**a**) Boundary conditions for sliding mode TENG. (**b**) Mesh quality deteriorates when Material 1 moving from state I to III. (**c**) The meshed geometry of sliding mode TENG.

The boundary conditions for the sliding model are illustrated in Figure 5a; Materials 1 and 2 have similar configurations as those for the contact mode TENG with an air buffer layer (the 1st air buffer layer) between them. In addition, an extra 2nd air buffer layer is introduced in the model which surrounds Material 1 as shown in Figure 5a. The 2nd air buffer layer can be divided into three regions: the ones on the left and right sides of Material 1, and the ones above and below Material 1. The air buffer layers above and below Material 1 are meshed with fixed mesh nodal points and the meshes move together with Material 1 during sliding movements, while those meshes in the air buffer layers on the left and right sides of Material 1 deform freely following the rules governed by Formula (6); they expand on one side and shrink on the other side accordingly, with the mesh nodal points remained unchanged when Material 1 moves horizontally, thus eliminating the topology-change issue. The moving mesh of the sliding mode TENG model at different times is illustrated in Figure 5b, showing that the meshes in the 2nd air buffer layer of the left side expand and those on the right side shrink when Material 1 moves from state I to III which allows an unchanged topology to be achieved.

As emphasized by the black dashed lines in state I of Figure 5b, the large translational motion in the sliding mode TENG will lead to mesh discontinuity (i.e., mesh nodal points discontinued), which happens between top air and 2nd air buffer, as well as between 1st and 2nd air buffer. The mesh discontinuity will lead to failure of calculation when the electrostatic constitutive equations are applied for modelling. Here, we introduce the charge continuity condition to mitigate the problem as schematically shown in Figure 5a. The charge density remains identical and continuous along the moving interfaces no matter where Material 1 moves.

As shown in state III of Figure 5b, the mesh quality deteriorates significantly when Material 1 leaves Material 2, which is different from that of the contact mode TENG simulation due to asymmetric geometry and movement. Therefore, mesh density and air buffer thickness optimization are necessary for accurate simulation.

The simulation parameters for mesh density and air gap thickness optimizations are listed in the Supplementary Information, Table S5. The definitions for the air gap and mesh density are illustrated in Figure 5c. The air gap thickness represents the distance between two tribo-materials, similar to that for the contact mode TENG simulation. The mesh density for the sliding mode TENG is varied by changing the mesh nodal points on the corresponding horizontal boundaries, which are marked by yellow circles in Figure 5c. Although thinner air gap thickness and denser mesh nodal points will result in more accurate simulation results, it takes much more simulation resources; therefore, a trade-off is needed.

**Figure 6:**
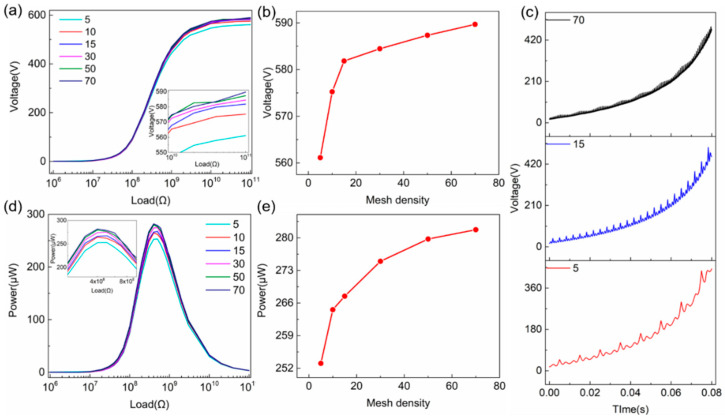
The simulated (**a**) voltage and (**d**) power output with increasing mesh density. (**b**) The summarized peak voltage at a 100-GΩ external load with increasing mesh density. (**e**) The summarized peak power output with increasing mesh density. (**c**) The voltage curve under different mesh density.

For the optimization of mesh density, voltage output under different loads with various mesh densities is shown in Figure 6a. The mesh density is determined by the number of the mesh nodal points at specific boundaries as the yellow circles marked in Figure 5c. It is clear that denser meshes can lead to higher voltage output as summarized in Figure 6b, which is obtained using a 100-GΩ external load. Moreover, with denser meshes, the voltage curve over time becomes smoother, which experiences more spikes with smaller amplitude as shown in Figure 6c. The effect of mesh density on power output is shown in Figure 6d. Although the optimal load remains unchanged for different mesh densities, the peak power slightly increases with denser meshes, as shown in Figure 6e.

**Figure 7:**
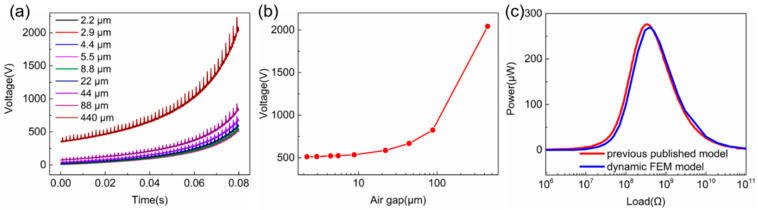
(**a**) The simulated voltage output and (**b**) summarized peak voltage under a 100-GΩ external load. (**c**) The comparison of calculated results based on dynamic FEM model and previous published model, demonstrating excellent consistency.

The voltage output under an open-circuit condition (100 GΩ) with different air gap thicknesses is shown in Figure 7a. The spiked voltage outputs under movement are attributed to the limited mesh density applied for less simulation time. As summarized in Figure 7b, the open-circuit voltage at different air gap thicknesses increases dramatically from 511.3 V to 2042.4 V when the air gap increases from 2.2 μm (1% of material thickness) to 440 μm at 0.08 s. If a 5% simulation discrepancy in voltage can be tolerated, the optimal thickness of air gap can be set to ~8.8 μm for an accurate simulation and relatively shorter calculation time.

For accurate simulation and a reasonable simulation time, an air gap thickness of 11 μm and base horizontal mesh nodal points of 60 are then chosen for the following simulation. The sliding mode TENG results based on the analytical calculation, that have already been verified with experimental results [23], are used to compare with our simulation results as depicted in Figure 7c, showing excellent consistency between them, demonstrating the high accuracy of the dynamic FEM model. It should be noted that in this analytical calculation and numerical simulation, the tribo-materials of the sliding mode TENG are not separated totally because the analytical method is unable to deal with the total separation situation.

**Figure 8:**
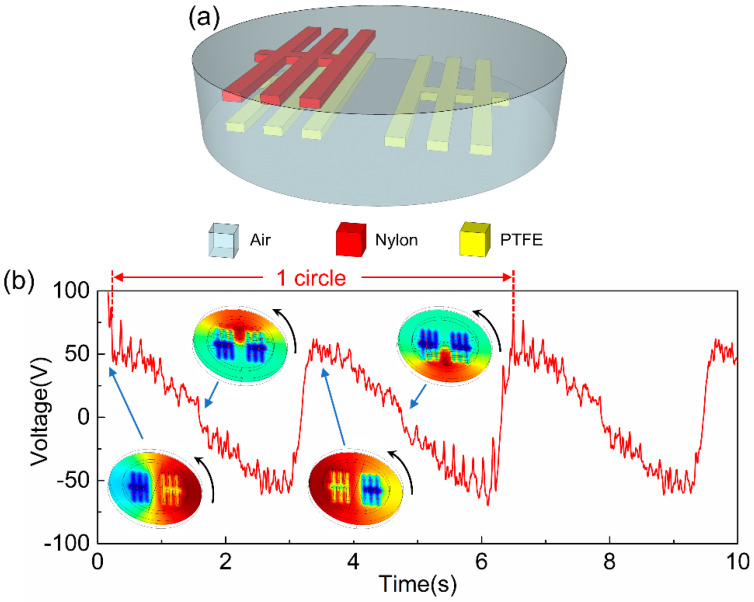
(**a**) The illustration of a 3D disk TENG with a bar type tribo-material. (**b**) The voltage output and electric potential distribution under a 50-MΩ external load.

For better demonstration of the advantages of the dynamic FEM model, a 3D rotating-type TENG is simulated as shown in Figure 8a, which utilizes nylon and PTFE as arrayed bar type tribo-materials in air atmosphere. The PTFE is fixed and nylon rotates anticlockwise. The voltage output under a 50-MΩ external load is shown in Figure 8b, which varies periodically as the top tribo-layer rotates. Inset images illustrate the top tribo-bar position and the electric potential distribution between two tribo-materials over one rotation. This clearly shows the capability of calculating complex 2D/3D geometries dynamically, demonstrating the superiority of our dynamic FEM model. Multiple spikes in the voltage output curve are generated when the arrayed bar type tribo-material approaches and leaves.

### 3.3. Design of TENG Performance by the Dynamic FEM Model

As shown above, the dynamic FEM model can be used to simulate the output power of TENGs as power sources as did by most of the previous work. Here, we will show the extra capability of the dynamic FEM model which can be utilized for designing a TENG with different performance/properties or a new type of device for sensing application.

**Figure 9:**
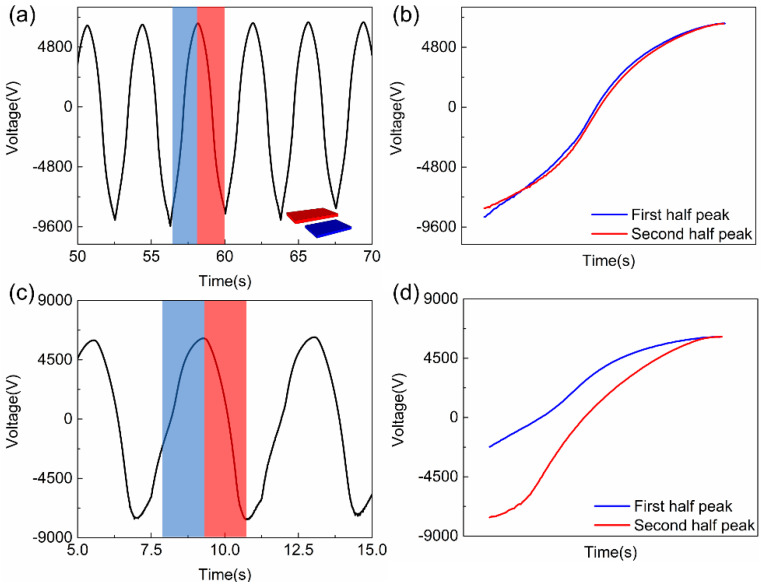
The voltage output of the rectangular patterned TENG under external load of (**a**) 10^12^ and (**c**) 10^11^ Ω. The voltage output of one cycle under external load of (**b**) 10^12^ and (**d**) 10^11^ Ω, demonstrating the voltage change of rectangular patterned TENG under lower external load and can be used in sensing applications to decide whether the moving tribo-material is under approaching or leaving state.

Rectangular shapes are commonly used for most TENG-based sensors owing to the easiness of design and fabrication, we will show that the dynamic FEM model allows us to design a triangular-shaped TENG sensor with better performance. The geometry of a common TENG is shown in inset of Figure 9a used in our FEM model. Material 1 (blue) is fixed, and Material 2 (red) is sliding forward and backward laterally. Figure 9a shows the output voltage of the TENG with an external load of 10^12^ Ω (near an open circuit condition), and the dynamic process can be seen in the Supplementary Information, Movie S1. The peak and valley voltage outputs occur when two materials are fully overlapped and separated, respectively. It is clear that the voltage output curves are highly symmetric, i.e., the voltage curves are identical when the two materials approach to and leave from each other. Figure 9b is the comparison of the two halves of a voltage output pulse. The blue curve represents the voltage output when the two materials move from the most separated position to the fully overlapped position, and the red curve represents the voltage output under opposite movement.

However, when the external load decreases to 10^11^ Ω, the shape of voltage output curve is deformed, as shown in Figure 9c. Although the peak voltage still occurs when the two materials are fully overlapped, the voltage output curves are significantly different when the two materials approach to (blue) and leave (red) from each other, as shown in Figure 9d. The discharge effect of the external load is severer when the two materials separate from each other. This can be utilized to design a sensor to detect whether the object is moving away or approaching. However, due to the symmetric structures of the two tribo-plates, it is still not possible to distinguish whether the object (i.e., the tribo-material) is moving from the leftmost or rightmost position as shown in Figure 9a,c, as the curve shapes of every cycle are exactly the same.

**Figure 10:**
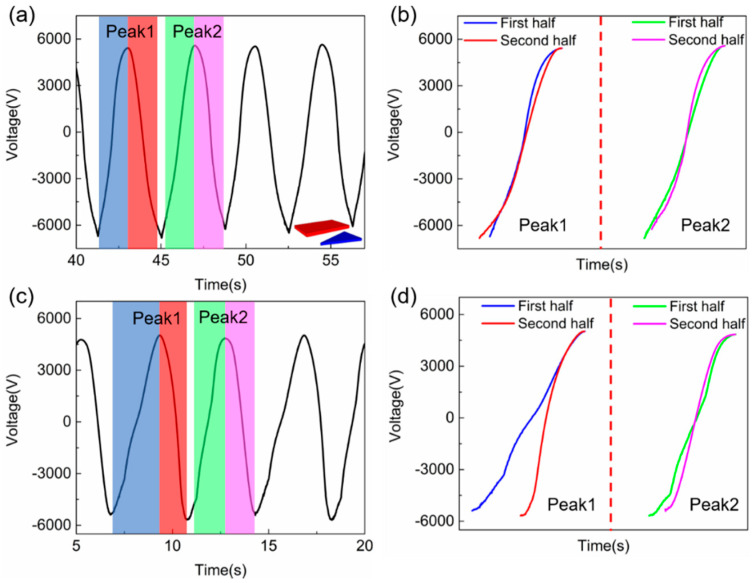
Voltage output of the triangular patterned TENG under an external load of (**a**) 10^12^ and (**c**) 10^11^ Ω. Voltage output of two different cycles under an external load of (**b**) 10^12^ and (**d**) 10^11^ Ω, demonstrating the voltage curve of triangular patterned TENG contains more information than that of rectangular patterned TENG, and the dynamic FEM model can be used to optimize the TENG tribo-material shape in sensing applications for multi-functional purposes.

Through the simulations using the dynamic FEM model, the shape of the tribo-plate is found to change the shape of voltage output curves, so that the approaching from different directions can be distinguished as shown in Figure 10a inset. All the simulation configurations are the same as those in Figure 9 except that the fixed tribo-plate has a triangular shape. The output voltage curve under a 10^12^ Ω external load is shown in Figure 10a, and the dynamic process can be clearly seen in the Supplementary Information, Movie S2. The peak and valley voltage output still occur at almost fully overlapped and separated positions, respectively. However, the curve shapes for different period (Peak 1 and 2) are slightly different under the 10^12^ Ω load as shown in Figure 10b. For a clearer demonstration, the voltage output under a 10^11^ Ω is shown in Figure 10c and the voltage output curves are shown in Figure 10d when the upper tribo-plate approaches from the leftmost position (blue), leaves to the rightmost position (red), approaches from the rightmost position (green) and leaves to the leftmost position (magenta). The reason why curve shape changes in Figure 10b,d is that the charges transfer quicker under a smaller load, which means the charge flow (voltage output curve) is much more sensitive to structural shape of TENGs. When the upper tribo-plate approaching from rightmost or leftmost position, it will experience different incoming shapes of the lower triangular tribo-plate, and the charge flow can sense the subsequent electric potential distribution change better due to lower eternal load. It is clear that more information can be obtained from the voltage output curves by TENG shape optimization, which can easily distinguish the moving situations. All the results have proven that the dynamic FEM model can accurately simulate the time-varying performance of TENGs with complex structures. Furthermore, based on this universal FEM model, the genetic algorithm can be applied to automatically design the TENG structure for tuned output curve in multi-functional sensing applications, which is under research.

## 4. Conclusions

A universal dynamic FEM model has been proposed, which is capable of simulating TENGs with complex 2D/3D geometries for time-varying electric outputs. A FEM model based on an air buffer layer and moving mesh method are used to eliminate the topology-change issues during simulation with practical movements. Then, the dynamic FEM model is systematically optimized with different air buffer thicknesses and mesh densities. The simulated results are compared with the experimental data and calculating results based on other methods, showing excellent consistency. A 3D disk TENG with a bar type tribo-plate is dynamically simulated to further demonstrate the capability of calculating complex 3D TENG geometries. Moreover, a dynamic output simulation based on rectangular and triangular shaped tribo-plates has been carried out to present the possibility of purposely tuned TENG electric output curves for multi-functional sensing applications using the dynamic FEM model. All results show that the dynamic FEM model has the potential in assisting structural design of TENGs with practical experimental setups, as well as accelerating the proceeding of practical TENG applications in the future.

## Data Availability

The data that support the findings of this study are available from the corresponding author upon reasonable request.

## Supplementary Materials

The following are available online at https://www.mdpi.com/1424-8220/20/17/4838/s1, Table S1: Parameters used for contact mode simulation in cosine movement, Table S2: Simulation time under different air gap thickness. (Simulation is processed using parameters shown in Table S1 with 10 MΩ external load. The mapped mesh for vertical and horizontal boundary is 10 and 50, respectively.), Table S3: Parameters used for contact mode simulation in practical movement, Table S4: Fitted curve formulas for practical movement, Table S5: Parameters used for sliding mode simulation in cosine movement, Video S1: The rectangular shaped TENG dynamic process and voltage output under 1 TΩ load, Video S2: The triangular shaped TENG dynamic process and voltage output under 1 TΩ load.
